# Circulating tumour DNA to guide rechallenge with cetuximab plus irinotecan in *RAS/BRAF* wild‐type metastatic colorectal cancer: A nonrandomised clinical trial

**DOI:** 10.1002/ctm2.70827

**Published:** 2026-09-11

**Authors:** De‐Shen Wang, Wen‐Chuan Chen, Chao Ren, Feng‐Hua Wang, Zhi‐Qiang Wang, Hui‐Yan Luo, Feng Wang, Qiong Tan, Yu‐Hong Li

**Affiliations:** ^1^ Department of Medical Oncology Sun Yat‐sen University Cancer Center, State Key Laboratory of Oncology in South China, Collaborative Innovation Center for Cancer Medicine Guangzhou Guangdong P. R. China

**Keywords:** cetuximab rechallenge, circulating tumour DNA, metastatic colorectal cancer, *RAS/BRAF* wild type

## Abstract

**Background:**

Mutant subclones driving anti‐EGFR resistance may progressively decline after therapy cessation, potentially reversing resistance to EGFR‐targeted treatment. We therefore examined whether rechallenge with cetuximab plus irinotecan is active and tolerable in patients selected by circulating tumour DNA (ctDNA) testing, focusing on metastatic colorectal cancer (mCRC) with *RAS/BRAF* wild‐type (WT) status.

**Methods:**

This single‐arm, Phase II clinical trial enrolled patients between November 2019 and February 2025. Eligible patients had histologically confirmed *RAS/BRAF* WT mCRC at diagnosis, prior cetuximab‐based first‐line therapy with clinical benefit, radiographic progression while on cetuximab or within 3 months after stopping it, subsequent progression after at least 1 additional systemic therapy, a cetuximab washout period of at least 4 months, and ctDNA‐confirmed *RAS/BRAF* WT status before rechallenge. Each 2‐week cycle delivered intravenous cetuximab (500 mg/m^2^) together with irinotecan (180 mg/m^2^). The primary endpoint was the objective response rate (ORR). We additionally assessed progression‐free survival (PFS), overall survival (OS), and treatment‐related adverse events (TRAEs).

**Results:**

This study enrolled 36 patients. Partial response was seen in 6 patients (16.7%), stable disease in 10 (27.8%), and progressive disease in 16 (44.4%); the remaining 4 patients were unevaluable due to early loss to follow‐up. The ORR reached 16.7% (95% CI: 7.9–31.9), with a disease control rate of 44.4% (95% CI: 29.5–60.4). The median PFS and OS were 3.9 months (95% CI: 3.1–4.7) and 13.0 months (95% CI: 8.1–17.9). Multivariate Cox regression analyses identified screening time interval (STI) as an independent predictor of PFS (HR = 0.93 per additional month; 95% CI: 0.88–0.99). TRAEs of grade 3–4 were uncommon and clinically manageable.

**Conclusions:**

These findings suggest that ctDNA‐guided rechallenge with cetuximab plus irinotecan may offer benefit in *RAS/BRAF* WT mCRC, with STI potentially informing patient selection. Nevertheless, further validation in larger randomised trials is warranted.

**Trial registration:**

ClinicalTrials.gov Identifier: NCT04224415.

**Graphical Headlights:**

1. *RAS/BRAF* wild‐type status confirmed by ctDNA may identify candidates for cetuximab rechallenge.
2. A longer screening time interval was associated with improved progression‐free survival.
3. These hypothesis‐generating findings warrant validation in larger randomised trials.

## Graphical abstract

For patients with refractory mCRC and preserved *RAS/BRAF* wild‐type status, ctDNA‐guided rechallenge of cetuximab combined with irinotecan represents a viable therapeutic avenue, offering durable disease control without compromising tolerability. Our findings underscore that optimising the screening time interval is crucial for maximising therapeutic benefit.

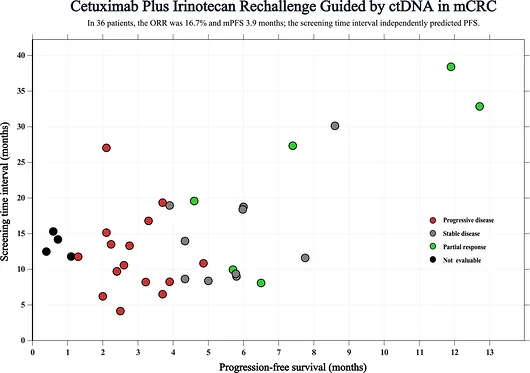

## 1 BACKGROUND

Globally, colorectal cancer (CRC) holds third place in cancer incidence and second in cancer‐related mortality. In China, the incidence of CRC has also risen sharply in recent years.^1^, ^2^ Among patients with CRC, 15%–20% have synchronous metastases at diagnosis, and 20%–50% of initially localised cases later develop metastases.^3^ For left‐sided *RAS/BRAF* wild‐type (WT) metastatic colorectal cancer (mCRC), the first‐line standard of care combines an anti‐EGFR antibody with chemotherapy.^3^, ^4^, ^5^ Although most patients initially respond to anti‐EGFR therapy, acquired resistance ultimately emerges and tumours progress. Nearly half of acquired resistance is caused by *RAS*, *BRAF*, or *EGFR*‐ectodomain mutations and *ERBB2* (*HER2*) or *MET* amplification, while other mechanisms include transcriptomic changes, genomic changes unrelated to innate anti‐EGFR resistance (such as *NF1*), and paracrine signaling.^6^, ^7^, ^8^, ^9^, ^10^, ^11^, ^12^ After failure of first‐line therapy, patients typically receive second‐line chemotherapy plus an antiangiogenic agent. For those who progress beyond second‐line treatment, subsequent options include bevacizumab plus Trifluridine/Tipiracil (FTD/TPI), fruquintinib, or regorafenib; however, the reported objective response rate (ORR) for these agents ranges from approximately 1% to 6%, while median progression‐free survival (PFS) remained 2–6 months.^13^, ^14^ Therefore, more effective treatment regimens need to be explored.

Theoretically, in patients with acquired anti‐EGFR resistance, resistant clones gradually decay during the treatment‐free interval, with an estimated half‐life of approximately 4 months, thereby creating a potential window for effective anti‐EGFR rechallenge.^15^, ^16^ Recent Phase II clinical studies and retrospective analyses have shown that anti‐EGFR rechallenge treatment is effective, but reports of response rates vary widely.^17^, ^18^, ^19^, ^20^, ^21^, ^22^, ^23^, ^24^ Previous studies have found that patients with *RAS/BRAF* WT status confirmed by circulating tumour DNA (ctDNA) benefit most significantly from this approach. Nonetheless, most of the data come from post‐hoc analyses with limited sample sizes or small‐scale prospective studies. Prompted by these considerations, we designed the present prospective study in 2019. Using ctDNA, we selected patients whose tumours remained *RAS/BRAF* WT and tested whether cetuximab plus irinotecan rechallenge was still effective and tolerable in this later‐line population. Here we present the results.

## 2 METHODS

### 2.1 Trial design and participants

In this prospective Phase II trial, patients whose ctDNA screening confirmed persistent *RAS/BRAF* WT received cetuximab plus irinotecan rechallenge on a single‐arm, open‐label basis. Before treatment initiation, ctDNA from all enrolled patients was profiled with the Bio‐Rad QX200 droplet digital PCR (ddPCR) platform. Key eligibility criteria included: aged 18–75 years; colorectal adenocarcinoma confirmed by histology; initially confirmed *RAS/BRAF* WT status; prior first‐line FOLFOX, FOLFIRI, or FOLFOXIRI plus cetuximab with a documented response (complete response [CR], partial response [PR], or stable disease [SD]) with PFS lasting at least 6 months; progression while receiving cetuximab or during the 3 months after its final dose; progression after second‐line or subsequent therapy; a cetuximab‐free interval of at least 4 months; one or more measurable lesions per RECIST version 1.1; confirmation of *RAS/BRAF* WT status via blood ctDNA testing; adequate haematological function; essentially normal coagulation, liver, and kidney function; a life expectancy exceeding 3 months; and written consent. Patients meeting any of the following were excluded: *RAS/BRAF* mutant status confirmed via blood ctDNA testing; another malignancy within the past 5 years; severe central nervous system diseases (e.g., brain metastases); other severe underlying medical conditions or complications; and allergy to any drug used in this trial. The Human Research Ethics Committee of Sun Yat‐sen University Cancer Center approved the study protocol (approval number: B2019‐150‐01). The trial was registered with ClinicalTrials.gov before enrolment (identifier: NCT04224415).

### 2.2 Treatment regimen and efficacy evaluation

Patients received cetuximab 500 mg/m^2^, together with irinotecan at 180 mg/m^2^. For patients with UGT1A1*28 7/7 or UGT1A1*6 A/A genotypes, as well as those with UGT1A1*28 6/7 or UGT1A1*6 A/G genotypes, the irinotecan dose was lowered to 150 mg/m^2^. Cycles were repeated every 2 weeks until progression, unacceptable toxicity, or withdrawal of consent. Imaging with contrast‐enhanced computed tomography or magnetic resonance imaging was performed at baseline and every four cycles, with responses graded per RECIST version 1.1.^25^ Toxicities were graded using the Common Terminology Criteria for Adverse Events, version 5.0 (CTCAE v5.0).

### 2.3 Outcomes and statistical analysis

The primary endpoint was ORR; secondary endpoints were PFS, overall survival (OS), and treatment‐related adverse events (TRAEs). Sample size followed Simon's optimal two‐stage design under a null ORR of 5%, an alternative ORR of 25%, a one‐sided type I error of 5%, and a type II error of 10%. Under these assumptions, 9 patients were required in the first stage. The trial would stop early if none of the first 9 patients responded. With at least one response, another 21 patients would be enrolled, for a total of 30. The regimen would be considered ineffective if ≤3 responses were observed. Accounting for a 15% dropout rate, at least 35 patients were planned for enrolment. As an exploratory analysis, a post‐hoc subgroup analysis based on the interval since last cetuximab treatment was conducted to explore predictive factors for cetuximab rechallenge combined with irinotecan. The screening time interval (STI) was defined as the period between the last anti‐EGFR and trial screening.^18^ For survival endpoints, Kaplan–Meier curves were constructed, and subgroup differences were examined in Cox proportional hazards models. All analyses were run in SPSS version 27 (IBM Corporation) and R version 4.6. All tests were two‐sided, with *p* < .05 taken as significant.

## 3 RESULTS

### 3.1 Patient characteristics

**FIGURE 1:**
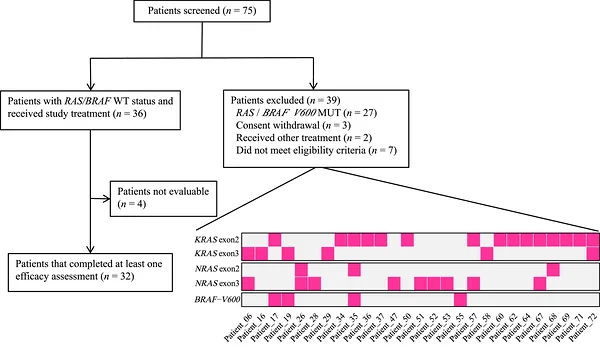
Study flowchart and molecular screening. WT, wild type; MUT, mutation.

Between 21 November 2019 and 25 February 2025, screening identified 75 patients; 27 patients were excluded due to detected mutations in ctDNA, and 12 did not enrol for other reasons, as detailed in Figure 1. Ultimately, 36 patients with confirmed *RAS/BRAF* WT status were enrolled and treated, and all of them entered the intention‐to‐treat (ITT) analysis. Baseline features of the 36 patients are shown in Tables 1 and S1. Of the 36 patients, 24 (66.7%) were male, and the median age at enrolment was 58 years (range: 29–73). The majority (*n* = 35, 97.2%) had an Eastern Cooperative Oncology Group Performance Status (ECOG PS) of 0 or 1. Left‐sided primary tumours were recorded in 35 patients (97.2%). Proficient mismatch repair or microsatellite‐stable status (pMMR/MSS) was observed in 33 patients (91.7%), with unknown status in 3. Synchronous metastases were present in 28 patients (77.8%). Metastatic sites included the liver (*n* = 31, 86.1%), lungs (*n* = 21, 58.3%), lymph nodes (*n* = 26, 72.2%), and peritoneum (*n* = 6, 16.7%). Meanwhile, 19 patients (52.7%) had metastases in more than two organs and a median of 3 previous treatment lines had been given (range 2–5). All had previously treated with cetuximab, oxaliplatin, and irinotecan, while 34 patients (94.4%) had also received bevacizumab. The median STI was 12.6 months (range: 4.1–38.4).

**TABLE 1:** Patient baseline characteristics.

Characteristics, *n* (%)	All patients (*n* = 36)
**Sex**	
Male	24 (66.7)
Female	12 (33.3)
**Age (years, median [range])**	58 (29–73)
**ECOG PS**	
0	21 (58.3)
1	14 (38.9)
2	1 (2.8)
**Site of primary tumour**	
Left colorectum	35 (97.2)
Right colon	1 (2.8)
**Mismatch repair status**	
Microsatellite stable	33 (91.7)
Microsatellite instable	0 (0.0)
NA	3 (8.3)
**Synchronous metastases**	
Yes	28 (77.8)
No	8 (22.2)
**Metastatic sites**	
Liver	31 (86.1)
Lung	21 (58.3)
Peritoneum	6 (16.7)
Lymph node	26 (72.2)
**Number of metastatic sites**	
≤2	17 (47.2)
>2	19 (52.7)
**Number of previous lines of treatment (median [range])**	3 (2–5)
**Previous treatment regimens**	
Cetuximab	36 (100)
Oxaliplatin‐based	36 (100)
Irinotecan‐based	36 (100)
Bevacizumab	34 (94.4)
**Screening time interval (months, median [range])**	12.6 (4.1–38.4)

Abbreviations: ECOG PS, Eastern Cooperative Oncology Group Performance Status; NA, not available.

### 3.2 Efficacy

**FIGURE 2:**
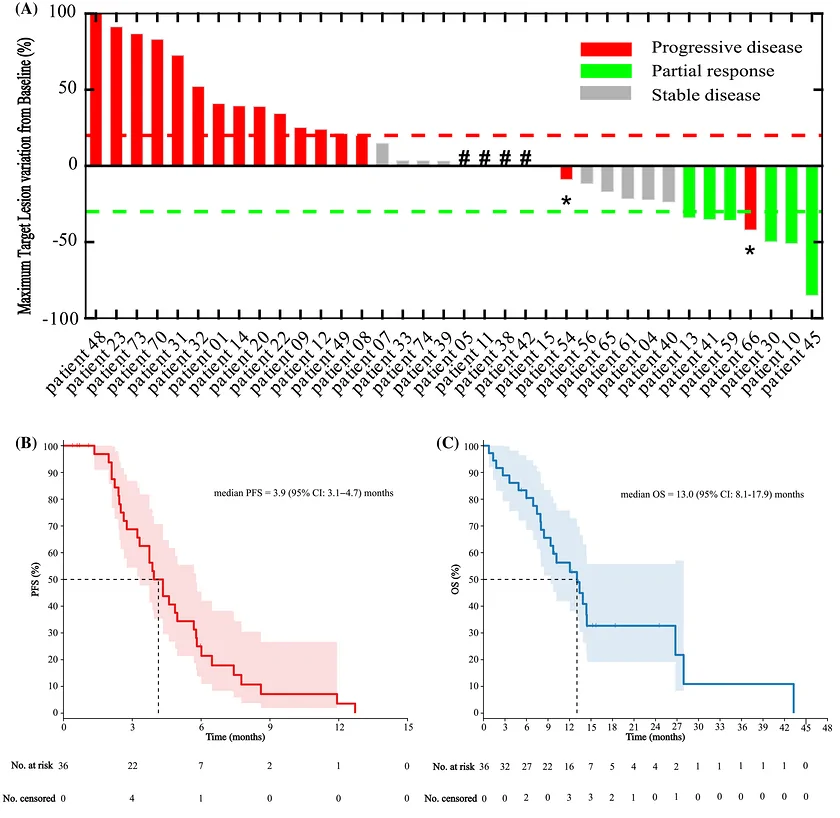
Tumour response and Kaplan–Meier estimates. (a) Waterfall plot showing the best responses in the target lesion according to RECIST version 1.1. Red, progressive disease; Grey, stable disease; Green, partial response; *, progressive disease due to new lesions; #, not evaluable. (b) Progression‐free survival (PFS). (c) Overall survival (OS).

At database lock (26 August 2025), follow‐up was a median of 15.7 months (95% confidence interval [CI]: 13.9–17.5); with one patient still on therapy. A total of 6 (16.7%) patients achieved PR, 10 (27.8%) had SD, 16 (44.4%) experienced progressive disease (PD), and 4 (11.1%) patients were not evaluable. The four non‐evaluable patients dropped out of follow‐up after one or two cycles of treatment. The primary endpoint was reached: among 36 intention‐to‐treat (ITT) patients, ORR and disease control rate (DCR) were 16.7% (95% CI: 7.9–31.9) and 44.4% (95% CI: 29.5–60.4). Median PFS and OS were 3.9 months (95% CI: 3.1–4.7) and 13.0 months (95% CI: 8.1–17.9) (Figure 2 and Table 2). Among the 32 evaluable patients, the sensitivity analysis found ORR and DCR values of 18.8% (95% CI: 8.9–35.3) and 50.0% (95% CI: 33.6–66.4).

**TABLE 2:** Tumour efficacy analysis.

Type of efficacy	ITT population (*n* = 36)
Best response, *n* (%)	
Complete response	0
Partial response	6 (16.7)
Stable disease	10 (27.8)
Progressive disease	16 (44.4)
Not evaluable	4 (11.1)
Objective response rate, *n* (%, 95% CI)	6 (16.7, 7.9–31.9)
Disease control rate, *n* (%, 95% CI)	16 (44.4, 29.5–60.4)
Progression‐free survival, median (95% CI), months	3.9 (3.1–4.7)
Overall survival, median (95% CI), months	13.0 (8.1–17.9)

Abbreviations: CI, confidence interval; ITT, intention to treat.

### 3.3 Subgroup analysis

**FIGURE 3:**
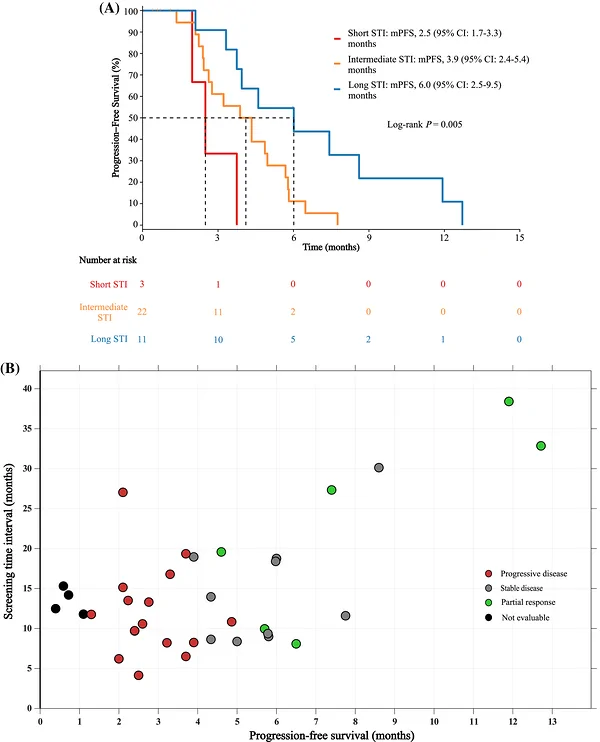
Exploratory Analysis of screening time interval. (a) Progression‐free survival categorised into three groups based on screening time interval. (b) The scatter plot illustrates the association between screening time interval and both progression‐free survival and tumour response. STI, screening time interval; mPFS, median progression‐free survival.

The association of clinical variables with PFS was evaluated in exploratory subgroup analyses (Table 3). In the primary analysis, STI entered the multivariate Cox model as a continuous variable and proved an independent predictor of PFS (HR = 0.93 per extra month of STI; 95% CI 0.88–0.99; *p* = .013). For exploratory illustration, STI was categorised into three groups based on the reported exponential decay half‐life of resistant clones (approximately 4 months), using twice this value as a threshold: short STI (< 8 months), intermediate STI (8–16 months), and long STI (> 16 months). PFS differed across the three STI groups (log‐rank *p *= .005). The median PFS was 2.5, 3.9, and 6.0 months in the short, intermediate, and long STI groups (95% CI: 1.7–3.3, 2.4–5.4, 2.5–9.5), respectively. Post‐hoc pairwise testing used a Bonferroni‐corrected significance level of 0.0167 to account for multiple comparisons. The long STI group achieved longer PFS than the short STI group (*p *= .008). After adjustment, neither the long‐versus‐intermediate comparison (*p *= .019) nor the intermediate‐versus‐short comparison (*p *= .079) reached significance (Figure 3). Median OS did not differ significantly between the short and long STI groups (13.4 months, 95% CI: 4.8–22.0 vs. 26.8 months, 95% CI: 6.6–47.0; *p* = .399).

**TABLE 3:** Univariate and multivariate analysis of progression‐free survival for patients who received rechallenge therapy.

			Progression‐free survival					
			Univariate analysis			Multivariate analysis		
Parameters	No. of patients	No. of events	HR	95% CI	*p*	HR	95% CI	*p*
Sex								
Female	12	8	1					
Male	24	23	2.04	0.81–5.12	.129			
Age (years)								
< 65	30	26	1					
≥ 65	6	5	0.69	0.24–2.01	.498			
ECOG PS								
0	21	19	1					
1–2	15	12	1.10	0.52–2.32	.804			
Synchronous Metastases								
No	8	5	1					
Yes	28	26	1.22	0.46–3.26	.692			
Liver metastases								
No	5	4	1					
Yes	31	27	2.14	0.64–7.19	.217			
Lung metastases								
No	15	13	1					
Yes	21	18	0.49	0.23–1.03	.061	1.17	0.44–3.08	.752
Peritoneal metastases								
No	30	26	1					
Yes	6	5	0.94	0.35–2.49	.899			
Lymph node metastases								
No	10	10	1					
Yes	26	21	1.22	0.56–2.67	.623			
Number of metastatic sites								
≤ 2	17	15	1					
> 2	19	16	0.81	0.39–1.66	.563			
Number of previous lines of treatment								
2	14	13	1					
≥ 3	22	18	0.47	0.22–1.00	.05	1.37	0.51–3.68	.528
Screening time interval (per month)			0.92	0.87–0.97	**.004**	0.93	0.88–0.99	**.013**

Abbreviations: CI, confidence interval; ECOG PS, Eastern Cooperative Oncology Group Performance Status; HR, hazard ratio.

Bold indicates statistical significance (*P* < 0.05).

### 3.4 Safety

Treatment‐related adverse events (TRAEs) were mostly skin rash (*n* = 23, 63.9%), diarrhoea (*n* = 14, 38.9%), alopecia (*n* = 13, 36.1%), anorexia (*n* = 11, 30.5%), increased aspartate transaminase (*n* = 10, 27.8%), fatigue (*n* = 10, 27.8%), and nausea (*n* = 9, 25.0%). Seven patients (19.4%) had grade 3–4 TRAEs: skin rash (*n* = 3, 8.3%), neutropenia (*n* = 3, 8.3%), anaemia (*n* = 2, 5.6%), and diarrhoea (*n* = 1, 2.8%) (Table S2). No patient stopped treatment because of toxicity. And the study treatment caused no deaths.

## 4 DISCUSSION

The present study found that ctDNA‐guided rechallenge therapy with cetuximab plus irinotecan produces meaningful activity with manageable toxicity in *RAS/BRAF* WT mCRC. Importantly, our findings align with those from the Ciardiello et al., CITRIC, PURSUIT, and PARERE studies, but differ considerably from the outcomes reported in studies such as Santini et al., CRACK, and FIRE‐4. The key characteristics and efficacy outcomes of these recent anti‐EGFR rechallenge studies are summarised in Table S3.^17^, ^18^, ^19^, ^21^, ^22^, ^23^, ^24^, ^26^, ^27^, ^28^, ^29^, ^30^, ^31^, ^32^, ^33^, ^34^

The ctDNA‐based patient selection strategies employed across anti‐EGFR rechallenge studies differ considerably. Several studies, including CRICKET, CAVE, VELO, and JACCRO CC‐08/CC‐09, performed retrospective ctDNA analyses and uniformly found worse PFS and OS when baseline *RAS/BRAF* mutations were present compared with *RAS/BRAF* WT.^17^, ^22^, ^23^, ^29^ The RASINTRO study prospectively selected *RAS/BRAF* WT via ctDNA and reported promising efficacy.^30^ Our findings are consistent with this body of evidence. More recently, some studies have explored broader ctDNA panels. CHRONOS and CITRIC incorporated *EGFR* extracellular domain (ECD) into their screening, in addition to *RAS/BRAF*, in an effort to further refine patient selection.^18^, ^26^ In a pre‐planned exploratory analysis, the CITRIC study used an ultraselected panel that included mutations in *RAS*, *BRAF V600E*, *EGFR* ECD, *MAP2K1*, and *PTEN*, as well as amplifications in *ERBB2* and *MET*. Patients with no alterations in this panel showed a trend towards longer mPFS compared with the gene‐altered group, though significance was not attained (*p* = .2).^26^ The PARERE study applied a similar hyperselection approach, screening for alterations in *AKT1*, *MAP2K1*, *EGFR*, *ERBB2*, non‐*BRAF‐V600E* class I/II, and *PIK3CA* exon 20. Post‐hoc exploratory analysis revealed greater benefit with panitumumab re‐treatment compared to regorafenib in the ctDNA negatively hyperselected population.^34^ These data indicate that while ultraselection is a promising direction, the incremental predictive value beyond RAS/BRAF remains to be validated in larger prospective trials. Serial ctDNA testing at multiple time points, as explored in the REMARRY and PURSUIT studies, may help refine patient selection.^27^ The assessment of low‐frequency resistance mutations represents another investigational strategy that warrants further study.^35^

This study identified the STI as an independent determinant of PFS in multivariate Cox model. Median PFS in the long STI group exceeded that in the short STI group (*p* = .008), and the short STI group showed no objective responses. Consistent with our findings, a longer screening time interval has been associated with improved rechallenge efficacy across multiple studies, including JACCRO CC‐08 study, the CRACK study, and retrospective analyses of cetuximab retreatment.^19^, ^21^, ^36^, ^37^ Notably, in PARERE, re‐treatment with panitumumab gave little survival benefit when the anti‐EGFR‐free interval was under 6 months.^34^ However, the CHRONOS trial did not observe an association between STI and treatment efficacy, suggesting that the relationship between STI and anti‐EGFR rechallenge outcome warrants further validation.^18^ Several limitations of the STI analysis should be noted: the three‐group classification was post‐hoc and based on a theoretical half‐life assumption, resulting in small subgroup sizes and insufficient power for pairwise comparisons after Bonferroni correction.

The treatment regimens used in anti‐EGFR rechallenge studies also vary substantially, including anti‐EGFR monotherapy, chemotherapy‐based combinations, immunotherapy‐based combinations, and other strategies. Herein, cetuximab was combined with irinotecan‐based chemotherapy, based on the synergy demonstrated in the BOND study.^38^ Conversely, the CHRONOS study investigated panitumumab monotherapy, the CAVE study evaluated cetuximab in combination with avelumab, the VELO study examined panitumumab plus Trifluridine/Tipiracil (FTD/TPI), and the CRACK study (Cohort B) assessed a triple combination of cetuximab, camrelizumab, and liposomal irinotecan.^17^, ^18^, ^19^, ^22^ The optimal rechallenge regimen remains to be established. Since bevacizumab plus FTD/TPI now represents the standard third‐line option,^14^ there are currently no studies on the head‐to‐head efficacy of anti‐EGFR rechallenge and FTD/TPI combined with bevacizumab. Accordingly, we initiated a randomised clinical trial of ctDNA‐guided cetuximab plus FTD/TPI rechallenge with bevacizumab plus FTD/TPI in *RAS/BRAF* WT refractory mCRC (NCT Number: NCT07012954).

Several limitations apply. First, the single‐arm design at a single centre, together with the modest sample (*n* = 36), constrained subgroup analyses and generalisability. Second, the post‐hoc three‐group STI classification and its associated statistical caveats should be considered when interpreting the exploratory STI analyses. Third, this study only screened for *RAS/BRAF* mutations. Other resistance alterations, such as *EGFR* ECD mutations and *ERBB2* or *MET* amplification, were not assessed.

## 5 CONCLUSION

In conclusion, ctDNA‐guided rechallenge with cetuximab plus irinotecan showed measurable activity and acceptable tolerability in patients with *RAS/BRAF* WT mCRC. Subgroup analyses further suggest that the screening time interval might guide patient selection to optimise cetuximab rechallenge outcomes. However, with a single‐arm design and a small sample, confirmation of these hypothesis‐generating findings awaits larger randomised trials.

## AUTHOR CONTRIBUTIONS

Dr. Yu‐Hong Li has full access to all data in this study and is responsible for the integrity of the data and the accuracy of the data analysis. De‐Shen Wang and Wen‐Chuan Chen contributed equally to this work. Yu‐Hong Li and De‐Shen Wang designed this study and wrote the protocol. Yu‐Hong Li, De‐Shen Wang, Wen‐Chuan Chen, Chao Ren, Feng‐hua Wang, Zhi‐Qiang Wang, Hui‐Yan Luo, Feng Wang, and Qiong Tan recruited patients. Yu‐Hong Li, De‐Shen Wang, Wen‐Chuan Chen, and Qiong Tan collected data and did the statistical analysis. Yu‐Hong Li, De‐Shen Wang, and Wen‐Chuan Chen verified all study data and wrote the initial manuscript. All authors reviewed and revised the manuscript and approved the final version of the manuscript.

## CONFLICT OF INTEREST STATEMENT

The authors declare that they have no known competing financial interests or personal relationships that could have appeared to influence the work reported in this paper.

## ETHICS STATEMENT

This study was approved by the Human Research Ethics Committee of Sun Yat‐sen University Cancer Center (approval number: B2019‐150‐01) and was conducted in accordance with the Declaration of Helsinki. All participants provided written informed consent prior to enrolment. The trial was prospectively registered at ClinicalTrials.gov (NCT04224415).

## Supporting information



Supporting Information

## Data Availability

Due to patient privacy protection and legal restrictions in China, we are unable to make the data publicly available. However, researchers who wish to use the raw data for scientific research purposes may apply for access from the database administrator. The data relevant to this study have been deposited in the research data platform operated by Sun Yat‐sen University (https://rdd.sysu.edu.cn/). For inquiries regarding access, please contact the administrator at yjsinfo@mail.sysu.edu.cn. Researchers seeking access to individual participant data that underlie the results reported in this article must submit a formal application to Sun Yat‐sen University, clearly stating the intended use of the data and addressing ethical considerations.
