# Supplementary material for: Circulating tumour DNA to guide rechallenge with cetuximab plus irinotecan in RAS/BRAF wild‐type metastatic colorectal cancer: A nonrandomised clinical trial
Source: Clin Transl Med. 2026 Sep 11;16(9):e70827. doi: 10.1002/ctm2.70827 (PMC13563332; doi:10.1002/ctm2.70827)
Supplement: Supplementary file 1 [file CTM2-16-e70827-s001.docx]

**Circulating Tumor DNA to Guide Rechallenge with Cetuximab Plus Irinotecan in *RAS/BRAF* Wild-Type Metastatic Colorectal Cancer: A Nonrandomized Clinical Trial**

**Supplementary Material**

**Supplementary Table 1**. **Previous and subsequent treatment regimens of patients.**

| **Characteristics** | ***n* = 36 (%)** |
| --- | --- |
| **Number of previous lines of treatment** | |
| 2 | 14(38.9) |
| 3 | 12(33.3) |
| 4 | 8(22.2) |
| 5 | 2(5.6) |
| **Previous treatment** **regimens** | |
| Cetuximab | 36 (100) |
| Oxaliplatin-based | 36 (100) |
| Irinotecan-based | 36 (100) |
| Bevacizumab | 34 (94.4) |
| FTD/TPI ± Bevacizumab | 3 (8.3) |
| Fruquintinib ± anti-PD-1 | 7 (19.4) |
| Regorafenib ± anti-PD-1 | 3 (8.3) |
| Crizotinib | 1 (2.8) |
| **Subsequent treatment regimens** |  |
| FTD/TPI ± Bevacizumab | 3 (8.3) |
| Fruquintinib ± anti-PD-1 | 10 (27.8) |
| Regorafenib ± anti-PD-1 | 5 (13.9) |
| Chemotherapy ± Bevacizumab | 5 (13.9) |
| Clinical Trial | 3 (8.3) |
| Fruquintinib+FTD/TPI | 1 (2.8) |
| FTD/TPI +Cetuximab | 1 (2.8) |
| HDACi + anti-PD-1 + Bevacizumab | 1 (2.8) |
| anti-PD-1 | 1 (2.8) |
| Lost to follow-up | 5 (13.9) |
| Patients without progression | 1(2.8) |

Abbreviations: PD-1, programmed cell death protein-1; HDACi, histone deacetylase inhibitor.

**Supplementary Table 2. Treatment-related adverse events.**

| **TRAEs** | **Number of patients (%)**  **(*n* = 36)** | |
| --- | --- | --- |
|  | **All Grades** | **≥ Grade 3 (%)** |
| Anorexia | 11 (30.5) | 0 |
| Nausea | 9 (25.0) | 0 |
| Vomiting | 6 (16.7) | 0 |
| Stomatitis | 5 (13.9) | 0 |
| Diarrhea | 14 (38.9) | 1 (2.8) |
| Constipation | 3 (8.3) | 0 |
| Fatigue | 10 (27.8) | 0 |
| Skin rash | 23 (63.9) | 3 (8.3) |
| Hand and Foot Syndrome | 3 (8.3) | 0 |
| Alopecia | 13 (36.1) | 0 |
| Neutropenia | 5 (13.9) | 3 (8.3) |
| Anemia | 6 (16.7) | 2 (5.6) |
| Thrombocytopenia | 2 (5.6) | 0 |
| Increased AST | 10 (27.8) | 0 |
| Increased ALT | 7 (19.4) | 0 |
| Increased bilirubin | 1(2.8) | 0 |
| Increased creatinine | 1(2.8) | 0 |

Abbreviations: AST, aspartate aminotransferase; ALT, alanine aminotransferase

**Supplementary Table 3. Characteristics and outcomes of recent anti-EGFR rechallenge trials in metastatic colorectal cancer**

| **Study name** | **Treatment regimen** | **No. of patients** | **Liquid biopsy** | **Response time of first-line anti-EGFR** | **Time from last anti-EGFR to PD** | **STI** | **ORR** | **mPFS** | **mOS** |
| --- | --- | --- | --- | --- | --- | --- | --- | --- | --- |
| Santini D. et al | CET + FOLFIRI or Irinotecan | 39 | NA | ≥ 6 months | NA | NA | 53.8 % | 6.6 months | NA |
| CRICKET | CET + Irinotecan | 28 (ITT) | Retrospective | ≥6 months | ≤4 weeks | ≥4 months | 21 % | 3.4 months | 9.8 months |
| JACCRO CC-08 | CET + Irinotecan | 34 | NA | ≥6 months | NA | NA | 2.9 % | 2.4 months | 8.2 months |
| JACCRO CC-09 | PAN + Irinotecan | 25 | NA | ≥6 months | NA | NA | 8.3 % | 3.1 months | 8.9 months |
| JACCRO CC-08 and 09 (Exploratory analysis) | CET or PAN + Irinotecan | 16 | Retrospective | ≥6 months | NA | NA | 0 % | 3.1 months | 8.9 months |
| CAVE | CET + Avelumab | 77 (ITT) | Retrospective | NA | NA | ≥4 months | 7.8 % | 3.6 months | 11.6 months |
| CHRONOS | PAN | 27 | Prospective | NA | NA | NA | 30 % | 16 weeks | 55 weeks |
| WJOG8916G | CET + Trifluridine/Tipiracil | 56 (ITT) | Retrospective | NA | NA | NA | 3.6 % | 2.4 months | 9.8 months |
| Mariani S. et al | Irinotecan + cetuximab/cetuximab monotherapy | 26 | Retrospective | NA | <1 month | NA | 25% | 3.5 months | 5.0 months |
| VELO | PAN + Trifluridine/Tipiracil vs. Trifluridine/Tipiracil | 62 (ITT) | Retrospective | NA | NA | NA | 9.7% vs. 0% | 4 vs 2.5 months | 11.6 vs 13.1 months |
| CRACK (Cohort B) | CET + camrelizumab + liposomal irinotecan | 16 (mITT) | NA | NA | NA | NA | 25% | 6.9 months | 15.1 months |
| RASINTRO | CET or PAN ± chemotherapy | 42 (RAS/BRAF WT) | Prospective | ≥4 months | NA | NA | 9.5% | 3.3 months | 7.9 months |
| PARERE | PAN vs. Regorafenib or the reverse sequence | 213 (ITT) | Prospective | ≥6 months | NA | ≥4 months | 16.0%vs 1.9% /0% vs. 17.4% | 4.1 vs. 2.4 months /2.7 vs. 3.7 months | NA |
| FIRE-4 | CET+FOLFIRI or Irinotecan vs. Investigator's choice | 87 | Prospective | ≥6 months | NA | NA | 26.7% vs. 11.9% | 5.8 vs. 4.6 months | 17.6 vs. 15.1 months |
| REMARRY & PURSUIT | PAN+ Irinotecan | 50 (FAS) | Prospective | NA | ≤2 months | ≥4 months | 14% | 3.6 months | 12 months |
| CITRIC | CET + Irinotecan vs. Investigator's choice | 58 (ITT) | Prospective | ≥16 weeks | ≤4 weeks | NA | 9.7% vs. 3.7% | 4.64 vs. 2 months | 11.3 vs. 7.3 months |

Abbreviations: CET, cetuximab; PAN, panitumumab; STI, screening time interval; ORR, objective response rate; mPFS, median progression-free survival; mOS, median overall survival; PD, progressive disease; mITT, modified intention-to-treat; FAS, full analysis set; ctDNA, circulating tumor DNA; WT, wild-type; NA, not available.
